# The RT-PCR identification and sequence analysis of Apple chlorotic leaf spot virus from apple cultivars in Jiaodong Peninsula, China

**DOI:** 10.1080/13102818.2014.908005

**Published:** 2014-07-08

**Authors:** Dechang Hu, Lei Wang, Xiaoman Jiang, Ning Wang, Liang Gu

**Affiliations:** ^a^ College of Life Science, Ludong University, Yantai, Shandong, P.R. China; ^b^ Fruit Institute, Yantai Academy of Agricultural Sciences, Yantai, Shandong, P.R. China

**Keywords:** apple, ACLSV, RT-PCR, sequence

## Abstract

A set of specific primer pairs was utilized to detect Apple chlorotic leaf spot virus (ACLSV) from seven different apple cultivars in Jiaodong Peninsula via reverse transcription polymerase chain reaction (RT-PCR), and the sequence of ACLSV genome was analysed. The results indicate that: (1) High-purity total RNA could be successfully isolated using plant RNA rapid extraction kit. The ratios of A_260_/A_280_ varied between 1.8 and 2.1. The fragmentation in agarose gel was good and the 28S and 16S bands were clear, which suggested that the extracted RNA had better quality and could be used for RT-PCR. (2) The amplified products by RT-PCR were approximately 220 bp, which showed the tested samples were infected by ACLSV in this study. (3) Sequencing analysis showed that the lengths of the target fragments were 217 bp, and the sequence identity rate ranged from 85.7% to 99.1% at the nucleotide level aligned with the corresponding sequences of other ACLSV strains in National Center for Biotechnology Information.

## Introduction

Biotic stresses caused by various pathogens, such as viruses and viroids, usually reduce the yields of fruit crops. These virus infections frequently occur as mixed infections on nursery and commercial fruit trees,[1,2] rendering economic losses of 30%–40%.[3] Apple chlorotic leaf spot virus (ACLSV) is an important virus due to the wide host range it has. ACLSV is spread mainly through horticultural practices like grafting and pruning. With the unscrutinized trades in the seedling market and the chronic orchard cultivation, a number of apple virus diseases attack the major apple producing areas, posing a great threat to the fruit industry. The presence of ACLSV has been reported in Jiaodong Peninsula of China.[4] Nurturing and cultivation of virus-free seedlings are fundamental measures to contain viral diseases because ACLSV cannot be controlled by pharmaceutical chemicals. ACLSV consists of numerous strains that produce different symptoms in sensitive fruit tree cultivars, whereas other strains are latent in many apple and pear cultivars.[5] Therefore, the fast and effective measures of detection are particularly important.

Traditional methods of virus detection include the biological indexing of plants and the conventional enzyme linked immunosorbent assay (ELISA) method. The former is slow, and the result is restricted with seasons. The sensitivity of the ELISA detection is still not high enough, and sometimes it yields misleading diagnosis due to non-specific reactions.[6] All certification and quarantine programmes include the testing of mother materials as an important step by using sensitive and reliable methods like reverse transcription polymerase chain reaction (RT-PCR).[7] RT-PCR has been shown to be more sensitive than ELISA for the detection of fruit viruses and has been used as an important tool for result confirmation.[8–10] In recent years, RT-PCR techniques have been used to detect the economically important and widespread viruses such as ACLSV, apple stem pitting virus (ASPV), apple stem grooving virus (ASGV), etc.[5,11–13] The present work utilized RT-PCR to assess seven apple accessions at the Jiaodong Peninsula orchard. The results would contribute to the molecular biology information of ACLSV and provide theoretical foundation for virus-free seedling cultivation in the Jiaodong region, China.

## Materials and methods

### Plant materials collection

In this experiment, leaves were collected from seven apple accessions at the Apple resource garden of Yantai Agricultural Science Research Institution, China. Yantai district lies in the east of the Jiaodong Peninsula and is a main apple-producing area. The number and names of samples are shown in Table 1.

**Table 1.** Number of samples and tissues collected in this study.

No.	Genotype	Tissue
1	Yantai Fuji 1	Leaf
2	Yantai Fuji 2	Leaf
3	Yantai Fuji 3	Leaf
4	Yantai Fuji 4	Leaf
5	Yantai Red General	Leaf
6	Yantai 2001	Leaf
7	Yantai Fuji 6	Leaf

### Isolation of total RNA

Total RNA was isolated according to the protocol of EASY spin plant RNA rapid extraction kit (Beijing Aide Lai Biotechnology Co. Ltd., China) with some modifications. Fifty milligrams of plant material was ground in liquid nitrogen, transferred into 3 mL of RNeasy Lysis buffer (RLT) extraction buffer (adding β-mercaptoethanol and 300 μL PLAN Taid) and then mixed vigorously for complete homogeneity. After incubation for 5 min at room temperature, samples were centrifuged at 4 °C at 12,000 r/min (Eppendorf Centrifuge 5430R, Germany) for 3 min. The supernatant was then transferred into the adsorption column RA (Beijing Aide Lai Biotechnology Co. Ltd., China), and subjected to centrifugation at 12.000 r/min for 1 min; then 700 μL were added to the protein solution RW1 eluting adsorption column (Beijing Aide Lai Biotechnology Co. Ltd., China) at room temperature for 30 s, 12,000 r/min for 30 s, then repeated once again. Finally, the liquid waste was discarded. The adsorption column RA was put in an RNase-free centrifuge tube, and 50 μL of RNase-free water was added to the adsorption film at an exactly intermediate portion and after incubation for 1 min at room temperature the sample was centrifuged at 12,000 r/min for 1 min to get the elution. Extracted RNAs were stored at −70 °C.

The total RNA was detected by using the trace spectrophotometer (TU-1810, China) absorbance measurements in order to detect the concentration and purity. The absorbance values of the RNA isolations at wavelengths A_230_, A_260_ and A_280_ nm were obtained. To detect the total RNA quality, 1.5% agarose gel was used.

### RT-PCR

RNA was reversely transcribed with reference to the kit instructions. cDNA was used for RT-PCR amplification. The sequences of RT-PCR primers were newly designed from the nucleotide sequence of the coat-protein-coding region of ACLSV. ACL-1: 5ʹ-GGTAGAGAGGCTCTATTCACATCTTG-3ʹ; ACL-2: 5ʹ-GGA GCTTTTCACCCCAGCAATTGG-3ʹ. RT-PCR amplification was carried out in a 25 μL reaction mixture: 1 × PCR buffer, 2.5 mmol/L MgCl_2_, 0.2 mmol/L dNTPs, 0.4 mmol/L primers ACL-1 and ACL-2, 1U *Taq* DNA polymerase and 1.5 μL cDNA. The amplification programme was: 94 °C for 5 min, 1 cycle; 94 °C for 30 s, 62 °C for 30 s, 72 °C for 30 s for 35 cycles and 72 °C extension for 10 min.[11] The amplified products were stored at 4 °C. PCR products were electrophoresed using 1.5% agarose gel, and the bands were visualized by Gene genius ultraviolet gel imaging system (SYNGENE, England).

### Sequencing and sequence analysis of ACLSV

DNA products obtained by PCR were sent to BGI-Tech Company (Shanghai, China) for sequencing. Database search was performed with Blast local alignment search tool (BLAST) programs at the National Center for Biotechnology Information (NCBI). DNASTAR software was used to carry out sequence alignment by CLUSTAL method.

## Results and discussion

### Quality and density of RNA

**Figure 1.**
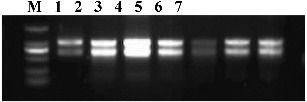
Electrophoretic analysis of total RNA of samples. The numbers 1–7 are listed in Table 1. M indicates DL2000 DNA marker.

The recorded ratios of A_260_/A_280_ and A_260_/A_230_ and RNA concentration are listed in Table 2. A_230_, A_260_ and A_280_ absorbance values were 0.033–0.090, 0.074–0.222 and 0.036–0.118, respectively. The A_260_/A_280_ was between 1.850 and 2.111, and A_260_/A_230_ between 1.805 and 2.654. The A_260_ value for the ‘Yantai Fuji 3’ sample was 0. 222 and the A_280_ value was 0.118, with an A_260_/A_280_ ratio of 2.467. The results about the RNA quality of the seven accessions tested through 1.5% agarose gel electrophoresis are shown in Figure 1. The 23S and 18S RNA bands were clear and separated completely, which indicated good quality of the total RNA. The results showed that the total RNA could meet the subsequent requirements for RT-PCR experiments.

**Table 2.** Absorbance values of A_230_, A_260_, A_280_, ratio of A_260_/A_280_, A_260_/A_230_ and concentration of the RNA isolations.

No.	A_230_	A_260_	A_280_	A_260_/A_280_	A_260_/A_230_	Concentration (μg/μL)
1	0.033	0.076	0.036	2.111	2.304	0.304
2	0.052	0.138	0.072	1.917	2.654	0.552
3	0.090	0.222	0.118	1.881	2.467	0.888
4	0.036	0.114	0.059	1.932	1.810	0.456
5	0.041	0.074	0.040	1.850	1.805	0.296
6	0.047	0.099	0.052	1.904	2.106	0.396
7	0.052	0.093	0.042	2.022	1.788	0.372

### Detection of RT-PCR product

**Figure 2.**
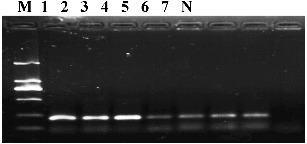
Amplification products indicative of the presence of Apple chlorotic leaf spot virus (ACLSV) (220 bp), obtained using RT-PCR for ACLSV detection in different samples. The numbers 1–7 are listed in Table 1. M indicates DL2000 DNA marker. N is the negative control.

The RNA of all samples was reversely transcribed, and PCR was performed using the designed primers ACL-1 and ACL-2. Double distilled water was taken as the negative control during the detecting process. The electrophoresis patterns showed that the length of the target fragment was about 200 bp, and no band was detected in the control lanes (Figure 2). The result indicated that the seven apple accessions were infected by ACLSV.

With high accuracy and sensitivity, RT-PCR technology would play an important role in the virus detections of fruit trees. The quality of RNA would directly affect the detection of reverse transcription and PCR amplification. In this study, RNA quality was shown to be good as detected by spectrophotometric measurements and agarose gel electrophoresis. The virus extraction protocol used here was easy to perform and yielded enough purified virus RNA from field tissue and PCR amplification was more reliable and stable.

### Molecular characterization of ACLSV isolations in apple

**Figure 3.**
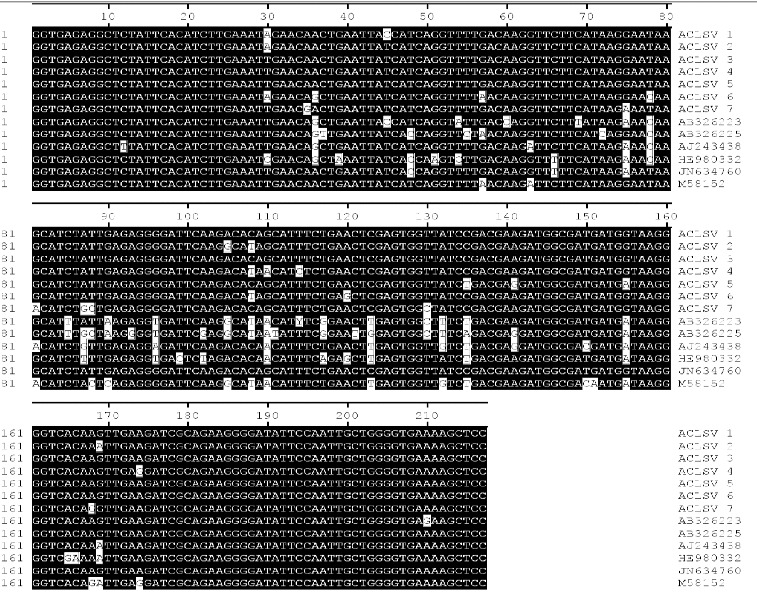
Alignment of the ACLSV sequences using the MegAlign program (DNASTAR) by the CLUSTAL method. Shaded (with solid black) residues are the nucleotides that match the consensus. ACLSV sequences obtained from GenBank are named as accession numbers.

The sequences of specific fragments obtained by RT-PCR were 217 bp in length. The ACLSV nucleotide sequences obtained in the present study were aligned with the corresponding sequences available in NCBI (Figure 3). The sequence identity rate ranged from 85.7% to 99.1% at the nucleotide level. This result showed that the seven samples investigated in this experiment had been infected with ACLSV.

More recently, a large number of studies were performed for biological characteristics and strain characteristics of ACLSV in different fruit trees.[5,14,15] However, little information on the molecular characterization was obtained for apple trees from the Jiaodong area of Shandong Province. Our research provided the ACLSV sequences by means of RT-PCR. There was no evidence for ACLSV varieties in this region. In the virus-free seedling cultivation processing, it is necessary to examine the virus transfer between the mother trees and seedlings. This study provides a theoretical basis for the virus identification in fruit trees of Jiaodong area, and virus quarantine, and would promise economic benefits for free-virus seedling production.

## Conclusions

In this study, high-purity total RNA could be successfully isolated using plant RNA rapid extraction kit. The amplified products by RT-PCR were approximately 220 bp, which suggested the tested apple samples in Jiaodong Peninsula were infected by ACLSV. Sequencing analysis showed that the lengths of the target fragments were 217 bp, and the sequence identity rate ranged from 85.7% to 99.1% at the nucleotide level aligned with the corresponding sequences of other ACLSV strains in NCBI. There was no evidence for ACLSV varieties in this region.

## References

[cit0001] Ismaeil F, Al-Jabor K, Myrta A, Mando MJ, Al-Saadoun E, Hassan M, Al-Chaabi S (2006). Viruses of pome fruit trees in Syria. OEPP/EPPO Bull.

[cit0002] Salem N, Mansour A, Al-Musa A (2005). Viruses of pome fruit viruses in Jordan. J Plant Pathol.

[cit0003] Cembali T, Folwell RJ, Wandschneider P, Eastwell KC, Howell WE (2003). Economic implications of a virus prevention program in deciduous tree fruits in the US. Crop Prot.

[cit0004] Su JM, Duan XJ, Yu Q, Sha YF, Li GC (2009). Preliminary report of Yantai city investigation and identification of the main fruit tree virus]. Shandong Agr Sci.

[cit0005] Wang LP, Hong N, Matic´ S, Myrta A, Song YS, Michelutti R, Wang GP (2011). Pome fruit viruses at the Canadian clonal genebank and molecular characterization of Apple chlorotic leaf spot virus isolates. Sci Hortic.

[cit0006] Hou YL, Zhang KC, Hu WY, Wu LP, Lin K, Yin SP (2002). Detection of several RNA viruses in fruit trees by RT-PCR. Chin J Virol.

[cit0007] Spiegel S, Thompson D, Varga A, Rosner A, James D (2006). Evaluation of reverse transcription-polymerase chain reaction assays for detecting Apple chlorotic leaf spot virus in certification and quarantine program. Can J Plant Pathol.

[cit0008] Kummert J (1998). Sensitive detection of apple stem grooving and apple stem pitting viruses from infected apple trees by RT-PCR. Acta Hortic.

[cit0009] Menzel W, Zahn V, Maiss E (2003). Multiplex RT-PCR-ELISA compared with bioassay for the detection of four apple viruses. J Virol Methods.

[cit0010] Spiegel S, Thompson D, Varga A, Rosner A, James D (2005). An Apple chlorotic leaf spot virus isolate from ornamental dwarf flowering almond (*Prunus glandulosa* ‘Sinensis’): detection and characterization. HortScience.

[cit0011] Hassan M, Myrta A, Polak J (2006). Simultaneous detection and identification of four pome fruit viruses by one-tube pentaplex RT-PCR. J Virol Methods.

[cit0012] Song YS, Hong N, Wang LP, Xu WX, Hu HJ, Tian R, Wang GP (2011). Molecular and serological diversity in Apple chlorotic leaf spot virus from sand pear (*Pyrus pyrilofia*) in China. Eur J Plant Pathol.

[cit0013] Zheng YY, Wang GP, Hong N, Song YS, You H (2007). Partial molecular characterization of Apple chlorotic leaf spot virus from peach and apple trees and prokaryotic expression for cp gene. Acta Phytopathol Sin.

[cit0014] Ana G, María JC, Gregorio B, Pedro D, Manuel R, Federico D, Pedro M, Hernández JA (2011). Changes in the antioxidative metabolism induced by Apple chlorotic leaf spot virus infection in peach [*Prunus persica* (L.) Batsch]. Environ Exp Bot.

[cit0015] Rana T, Chandel V, Hallan V, Zaidi AA (2009). Molecular evidence for the presence of Apple chlorotic leaf spot virus in infected peach trees in India. Sci Hortic.

